# Role of the growth step on the structural, optical and surface features of TiO_2_/SnO_2_ composites

**DOI:** 10.1098/rsos.181662

**Published:** 2019-01-09

**Authors:** Luca Rimoldi, Daniela Meroni, Eleonora Pargoletti, Iolanda Biraghi, Giuseppe Cappelletti, Silvia Ardizzone

**Affiliations:** 1 Dipartimento di Chimica, Università degli Studi di Milano, Via Golgi 19, 20133 Milano, Italy; 2 Consorzio Interuniversitario Nazionale per la Scienza e la Tecnologia dei Materiali (INSTM), Via Giusti 9, 50121 Firenze, Italy

**Keywords:** titanium dioxide, tin oxide, TiO_2_/SnO_2_ composite, hydrothermal treatment, calcination

## Abstract

TiO_2_/SnO_2_ composites have attracted considerable attention for their application in photocatalysis, fuel cells and sensors. Structural, morphological, optical and surface features play a pivotal role in photoelectrochemical applications and are critically related to the synthetic route. Most of the reported synthetic procedures require high-temperature treatments in order to tailor the sample crystallinity, usually at the expense of surface hydroxylation and morphology. In this work, we investigate the role of a treatment in an autoclave at a low temperature (100°C) on the sample properties and photocatalytic performance. With respect to samples calcined at 400°C, the milder crystallization treatment promotes anatase phase, mesoporosity and water chemi/physisorption, while reducing the incorporation of heteroatoms within the TiO_2_ lattice. The role of Sn content was also investigated, showing a marked influence, especially on the structural properties. Notably, at a high content, Sn favours the formation of rutile TiO_2_ at very low reaction temperatures (100°C), thanks to the structural compatibility with cassiterite SnO_2_. Selected samples were tested towards the photocatalytic degradation of tetracycline in water under UV light. Overall, the low-temperature treatment enables to tune the TiO_2_ phase composition while maintaining its surface hydrophilicity and gives rise to well-dispersed SnO_2_ at the TiO_2_ surface.

## 1. Introduction

TiO_2_/SnO_2_ composites find application in numerous cutting-edge fields, such as fuel cells [1], gas sensors [2,3] and photocatalysis [4,5]. Owing to the near isomorphism [6] of rutile TiO_2_ (*P*4_2_/mnm, *a* = 4.5937 Å, *c* = 2.9587 Å) and cassiterite SnO_2_ (*P*4_2_/mnm, *a* = 4.7382 Å, *c* = 3.1871 Å), TiO_2_/SnO_2_ composites can form stable heterojunctions exhibiting improved rate capacity and cycling stability as anodes in photoelectrochemical applications [7]. Moreover, the different work functions of the two oxides (4.9 and 4.2 eV for SnO_2_ and TiO_2_, respectively [8]) favour the occurrence of electron transfer from the TiO_2_ conduction band to the SnO_2_ one, giving rise to a contact potential at the interface [9]. This phenomenon is highly desirable in photoelectrochemical applications, such as photocatalysis [10], as it enhances charge carrier separation and, as a result, the device efficiency.

Numerous synthetic approaches have been reported for the synthesis of TiO_2_/SnO_2_ composites, including sol–gel [5,11], topotactic synthesis [12], chemical vapour deposition [13], impregnation [14], hydrothermal methods [15,16] and electrochemical deposition [17]. However, most of these synthetic procedures involve a final high-temperature treatment to promote crystallinity [3,5,11–14]. Also in the case of hydrothermal/solvothermal treatments, most of the reported procedures involve high temperatures (180–225°C) and, consequently, very high pressures [18–21]. The use of milder treatments and their role on the materials properties has been by far less investigated. Low-temperature crystal growths require less energy and can be performed *in situ* on a broad range of process-compatible materials, including heat-sensitive materials such as polymers. Moreover, such mild treatments can enhance the final properties of the prepared materials like the structural composition, morphology and surface hydroxylation. In particular, a higher degree of water chemi/physisorption at the oxide surface can result in a better stability of photocatalyst suspensions, which is beneficial for applications such as water pollutant remediation [22,23] as well as hydrogen production [24]. Moreover, surface hydroxyl groups can trap holes and hinder charge recombination [25].

In this work, we investigated the role played by a prolonged treatment (170 h) at a mild temperature (100°C) in an autoclave on the structural, morphological, optical and surface properties of TiO_2_/SnO_2_ composites prepared by a sol–gel procedure. In the literature, TiO_2_ growth by hydro/solvothermal methods requires shorter treatments (24–72 h) but in much harsher conditions [18–21], whereas flash treatments (up to few hours) are used only when crystalline TiO_2_ seeds are adopted [15,16]. Here, a prolonged autoclave treatment enabled to promote a slow crystal growth in less demanding conditions, hence without the need of expensive instrumentation and with much lower safety concerns. Samples prepared by this procedure will be compared to analogues obtained by a conventional calcination step at a high temperature. For the sake of comparability, both post-treatments were performed on the same purified xerogels; this solvent removal technique was selected due to its simplicity and widespread usage.

## 2. Material and methods

### 2.1. Sample preparation

Analytical grade reagents and solvents were purchased from Sigma-Aldrich and used without further purification. Doubly distilled water, passed through a Milli-Q apparatus, was used throughout the work.

Titanium(IV) isopropoxide (10.7 g) and 2-propanol (11.3 g) were mixed for 15 min at 60°C. In the case of Sn-promoted samples, SnCl_4_·5H_2_O was added to the mixture in the appropriate amounts. Then, 65 ml of 0.29 M NH_4_OH aqueous solution was dripped into the reactor while stirring vigorously. The reaction mixture was then stirred for 90 min. The resulting precipitate was washed several times by centrifugation-resuspension cycles and later dried at 80°C overnight. Fractions (1.5 g) of the dried xerogel were suspended in 50 ml of water, transferred into a 100 ml Teflon-lined stainless-steel autoclave and treated for 170 h at 100°C.

The pristine and Sn-promoted samples were, respectively, labelled as Ti and TiSn*x*, where *x* represents the Sn/Ti molar ratio (5–20%). For the sake of comparison, a reference sample, named TiSn5_400, will also be presented: this sample was prepared by a sol–gel procedure identical to that of TiSn5, but it was then calcined at 400°C under O_2_ flux for 6 h, according to a previously reported procedure [5].

### 2.2. Material characterization

Samples were characterized by X-ray powder diffraction (XRPD) using a Philips PW 3710 Bragg-Brentano goniometer working with graphite-monochromated Cu K*α* radiation (40 kV, 40 mA). The instrument was equipped with a scintillation counter and 1° divergence slit, 0.2 mm receiving slit and 0.04° Soller slit systems. The phase quantification was performed by Rietveld refinement as implemented by the Quanto software. The average crystallite sizes of the anatase and rutile phases were estimated applying the Scherrer equation on the (101) and (110) peaks, respectively.

Specific surface areas were determined via N_2_ adsorption/desorption curves in subcritical conditions (Coulter SA3100) by elaboration with the Brunauer–Emmett–Teller (BET) method. Pore size distributions were determined from the desorption isotherms using the Barrett–Joyner–Halenda (BJH) method.

Diffuse reflectance spectra (DRS) were collected on a Shimadzu UV-2600 UV–vis spectrophotometer equipped with an integrating sphere. Spectral scans were performed in the 250–700 nm range, using BaSO_4_ as a reference. The apparent band gaps of the samples, *E_g_*, were determined according to the Kubelka–Munk equation.

Survey and high-resolution X-ray photoelectron spectra (XPS) were acquired on a Surface Science Instruments M-probe apparatus. The instrument employs monochromatic Al K*α* radiation (1.486 keV) with a spot size of 200 × 750 µm and a 25 eV pass energy. Spectra were referenced to C 1s at 284.8 eV in order to correct for charge build-up.

Energy dispersive X-ray (EDX) analyses were performed on a Hitachi TM 1000 scanning electron microscope.

Fourier-transform infrared (FTIR) spectra were collected in the 400–4000 cm^−1^ range on a PerkinElmer Spectrum 100 attenuated total reflectance (ATR) spectrometer.

### 2.3. Photocatalytic tests

The degradation of tetracycline hydrochloride (TC) in water was tested under UV irradiation (Jelosil HG500 halogenide lamp, effective power density 30 mW cm^−2^) on a previously reported photocatalytic set-up [5,23] at spontaneous pH (*ca* 4). Reactions were carried out under O_2_ bubbling, adopting a 35 ppm initial pollutant concentration and 0.5 g l^−1^ photocatalyst content. Before irradiation, the suspension was kept in the dark for 30 min to achieve adsorption equilibrium. Both the pollutant disappearance and the total mineralization were monitored by UV–vis spectroscopy and total organic carbon measurements, respectively, according to previously reported procedures [23]. Irradiation was continued for 180 min. Photolysis tests showed less than 5% mineralization in the absence of photocatalyst under irradiation.

## 3. Results and discussion

**Figure 1.**
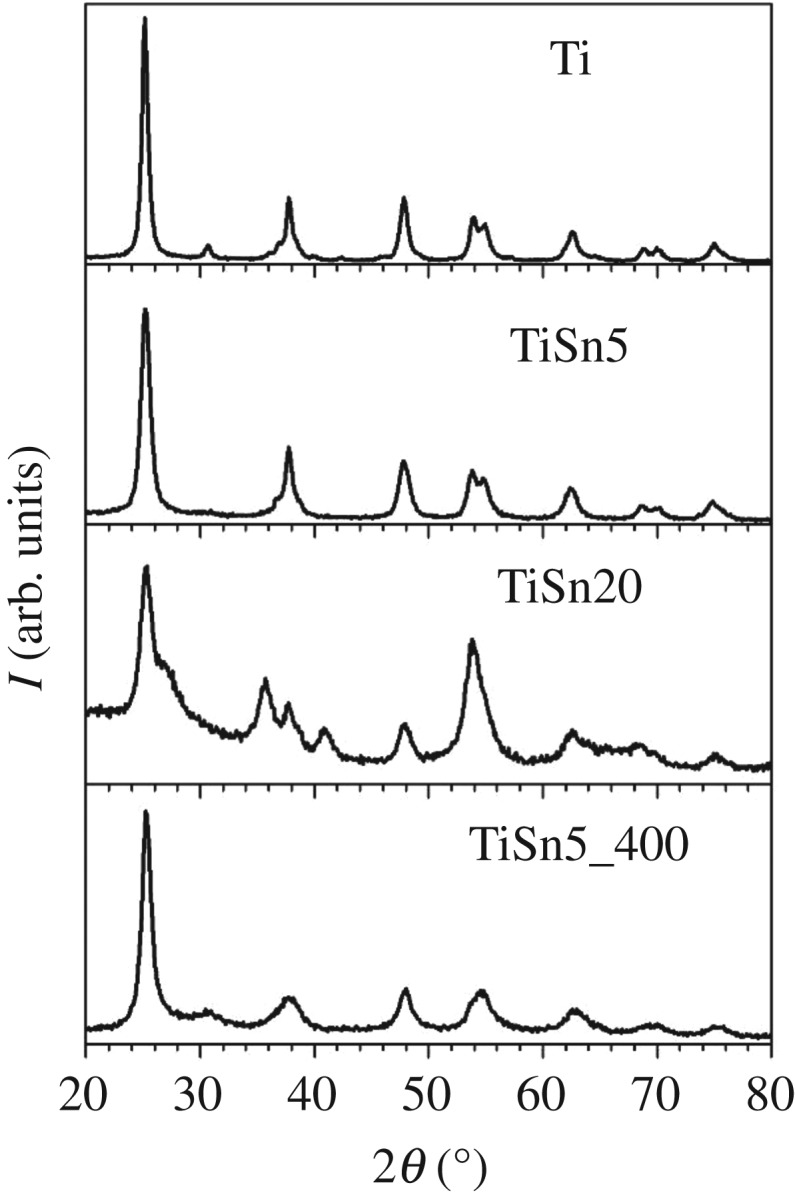
XRPD patterns of samples prepared with the low-temperature treatment. For the sake of comparison, the diffractogram of a calcined sample is reported as a reference, see [5].

Figure 1 reports the samples' XRPD patterns. Both the Sn content and the type of crystal growth treatment play a notable role of the phase composition. As appreciable from the peak at *2θ* ≈ 30.8° characteristic of the brookite (121) reflection, the Ti sample is an anatase–brookite mixture with the anatase polymorph in a large majority (table 1). The brookite peak almost disappears upon promotion with 5% Sn: the brookite amount decreases from 20% to about 4% (table 1). No peaks attributable to SnO_2_ phases can be detected. Interestingly, the calcined sample with the same Sn amount (TiSn5_400) shows instead an anatase/brookite ratio 80/20. Apparently, the mild, prolonged hydrothermal treatment in the presence of metal species favours the growth of the anatase polymorph. Wet and hydrothermal treatments of titanium salts or oxide precursors generally promote the formation of anatase [26,27], even in mixed solvents [28]. The formation of pure brookite by hydrothermal procedures has been reported but requires specific conditions and post-treatments (e.g. selective etching of anatase in HF solution) [29]. The presence of metal species during the hydrothermal growth may further promote the anatase phase by possible insertion of saline species in the oxide framework, as already suggested in the case of TiO_2_ aerogels [30].

**Table 1.** Physicochemical parameters of pristine TiO_2_ and TiO_2_/SnO_2_ composites: phase composition (A: anatase, B: brookite, R: rutile) and average crystallite size of the most abundant phase, *d*, estimated from XRPD analyses; Sn/Ti molar ratios from EDX analyses; specific surface area, *S*_BET_, and total pore volume, *V*_PORES_; apparent band gap values determined by Kubelka–Munk elaboration, *E_g_*. A calcined sample is reported as reference, see [5].

sample	phase composition (%)	*d* (nm)	Sn/Ti (EDX)	*S*_BET_ (m^2^ g^−1^)	*V*_PORES_ (ml g^−1^)	*E*_g_ (eV)
Ti	80A–20B	16	—	110	0.400	3.32
TiSn5	96A–4B	9	4	163	0.341	3.24
TiSn20	30A–70R	7	23	163	0.322	3.31
TiSn5_400	78A–22B	9	4	215	0.079	3.11

The Rietveld refinement of XRPD lines in the case of TiSn20 is complicated by the proximity of the most intense reflections of the three involved polymorphs (anatase TiO_2_ (101) at 2*θ* = 25.4°; rutile TiO_2_ (110) at 2*θ* = 27.5°; cassiterite SnO_2_ (110) at 2*θ* = 26.6°) and by the peak broadness. The fitting procedure yields rutile TiO_2_ as the most abundant phase in the presence of anatase in a ratio which can be roughly estimated to be anatase/rutile 30/70. The formation of rutile TiO_2_ in such mild conditions is not usual and in the present case can be traced back to the crystalline habit similarity between rutile TiO_2_ and cassiterite SnO_2_ (space group: *P*4_2_/mnm; tetragonal structure) [6]. However, even at this high Sn content, no crystalline segregated cassiterite SnO_2_ can be appreciated.

The used sol–gel approach gives rise, for both pristine [31] and Sn-promoted TiO_2_ [4,5], to polycrystalline particles with nanometric crystalline domains, in good agreement with the present XRPD findings. Previous HR-TEM investigations have shown, on the grounds of both interplanar distances and FFT patterns, that different phases coexist on single particles [4,5,31].

With respect to the Ti sample, the presence of Sn in the synthesis invariably lowers the average crystallite size (table 1). This is possibly the result of a prevailing surface location of the metal species, which can inhibit the crystal growth. This occurrence, together with the lack of appreciable crystalline phases related to Sn compounds, has prompted us to analyse the surface composition of Sn-promoted samples by XPS.

**Figure 2.**
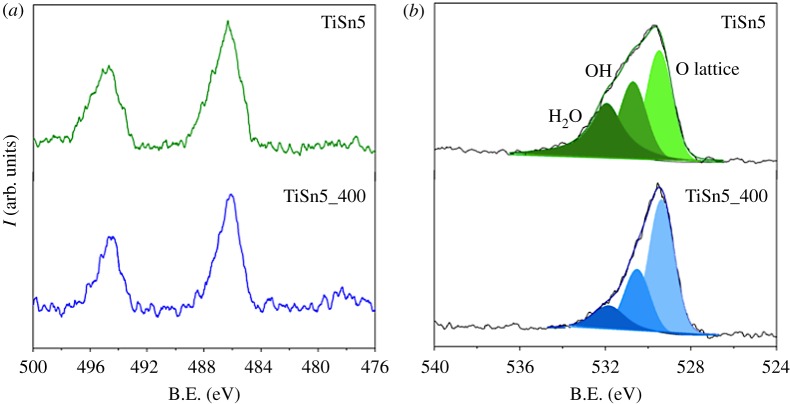
XPS spectra of TiSn5: Sn 3d region (*a*) and O 1s region, with relative fitting (*b*). For the sake of comparison, the spectra of TiSn5_400 are reported, see [5].

Figure 2 reports the comparison between the Sn 3d (*a*) and the O 1s (*b*) regions of TiSn5 and the calcined analogue, TiSn5_400. The Sn 3d region (figure 2*a*) of both samples presents the Sn 3d_5/2_ and Sn 3d_3/2_ doublet at 486.2 and 494.6 eV, respectively, indicative of Sn(IV) species [32]. The Sn/Ti atomic ratios obtained by XPS determinations (17% and 7% for TiSn5 and TiSn5_400, respectively) were larger than the relative values obtained by EDX analysis (table 1, electronic supplementary material, table S2); the latter were comparable to the stoichiometric amounts, within the analytical limits of the technique (electronic supplementary material, figure S2). Owing to the surface-sensitive nature of XPS, this observed difference supports the prevailing location of the Sn species at the surface of the material, where they can thwart the crystallite growth. The Sn/Ti atomic ratio of sample TiSn5 is more than twice that of TiSn5_400 (electronic supplementary material, figure S1), suggesting a much lower bulk penetration of metal species during the low-temperature growth.

XPS spectra of the O 1s region (figure 2*b*) show very broad peaks, supporting the presence of more than one oxygen species. The fitting procedure of the O 1s spectral components, based on binding energy (B.E.) values presented in the literature [33], showed the presence of three components. The main one, at lower binding energies (529.4 eV), can be assigned to reticular O in the oxide; a second component at 530.6 eV can be attributed to surface OH groups, while the component at the highest B.E. (531.9 eV) can be related to chemisorbed water [33]. The fitting procedure gave rise, for the two samples, to B.E. values coinciding within the experimental error. Notably, the O 1s peak of TiSn5 presents a much more appreciable shoulder at high B.E. with respect to the calcined analogue, which the fitting attributes to a much larger content of chemisorbed water (components intensity ratio: H_2_O/O_lattice_ = 0.90 and 0.21 for TiSn5 and TiSn5_400, respectively; electronic supplementary material, table S1).

**Figure 3.**
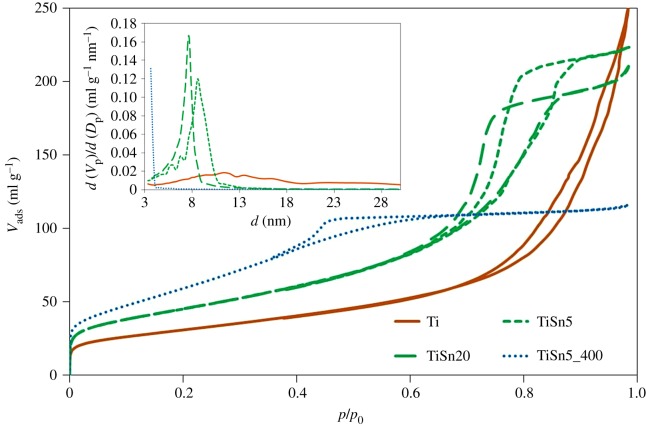
N_2_ adsorption–desorption isotherms in subcritical condition (−196°C) and pore size distribution (inset) of pristine TiO_2_ and TiO_2_/SnO_2_ samples. The curves of a calcined sample are reported for the sake of comparison, see [5].

Figure 3 reports the N_2_ adsorption–desorption isotherms in subcritical conditions of the samples, which all show a mesoporous character (electronic supplementary material, figure S3). The shape of the hysteresis loop varies remarkably between the metal containing samples and the Ti sample. The Ti sample presents an H3-type hysteresis loop, characteristic of slit-shaped pores, while the loops of the Sn-promoted samples are typical of bottleneck pores. Moreover, samples differ notably for their pore size distributions (figure 3 inset), as shown by the shift in the hysteresis loop on the pressure axis. By the sequence of the monolayer knee, the specific surface area is observed to increase upon metal promotion in passing from Ti to TiSn5 (figure 3 and table 1). The calcination treatment promotes a further increase in specific surface area (table 1), possibly due to a larger fraction of smaller pores freed by the thermal oxidizing procedure (figure 3 inset). By comparing the morphological features of TiSn5 and TiSn5_400, a much larger pore volume is appreciable for the former sample (table 1), as a consequence of the larger average pore size (figure 3 inset). This is noteworthy considering that both samples were prepared from the same xerogel and this solvent removal procedure is known to reduce the degree of porosity due to the curvature in the concave menisci of the flooded gel network, which provokes enhanced evaporation and ensuing pore collapse [30]. The increase in the Sn amount from 5 to 20% does not modify appreciably the surface area and total pore volume (figure 3), mainly giving rise to a slight decrease in the average pore size (figure 3 inset).

**Figure 4.**
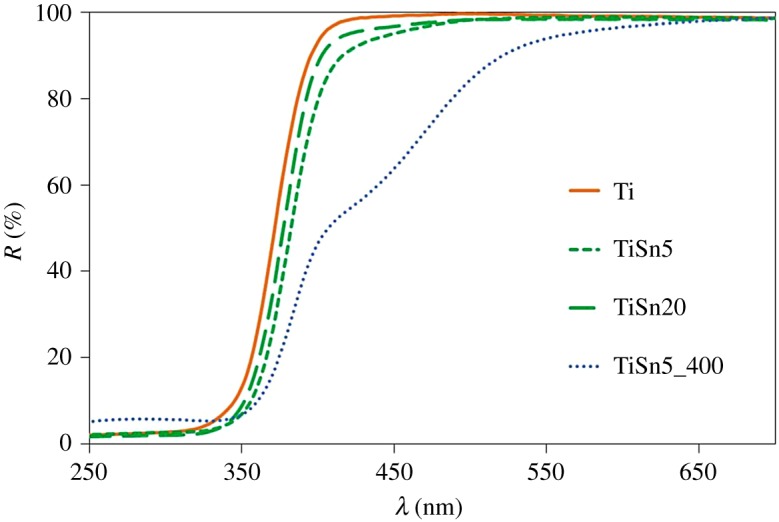
DRS of pristine TiO_2_ and TiO_2_/SnO_2_ samples; the spectrum of TiSn5_400 is reported as reference, see [5].

The optical properties of the samples were investigated by DRS (figure 4). Both the Ti and the TiSn*x* samples show typical profiles of bare TiO_2_ samples. The Sn addition does not give rise to clear trends, also in terms of the apparent band gap (table 1, electronic supplementary material, figure S4). Conversely, the calcined sample presents a visible light absorption at around 450–500 nm, often reported in N-doped samples and attributed to the formation of mid-gap levels ensuing from N bulk doping [10,34,35]. The visible absorption of the calcined sample can thus be traced back to N-doping of the oxide lattice due to the NH_4_OH added during synthesis. The notable difference in the light absorption of TiSn5 and TiSn5_400 proves that the presently adopted mild growth conditions do not promote the formation of bulk colour centres ensuing from N species lattice incorporation. In this respect, FTIR determinations were performed to gain further insight into the location of N species, deriving from the synthetic procedure, in Ti and TiSn*x* samples.

**Figure 5.**
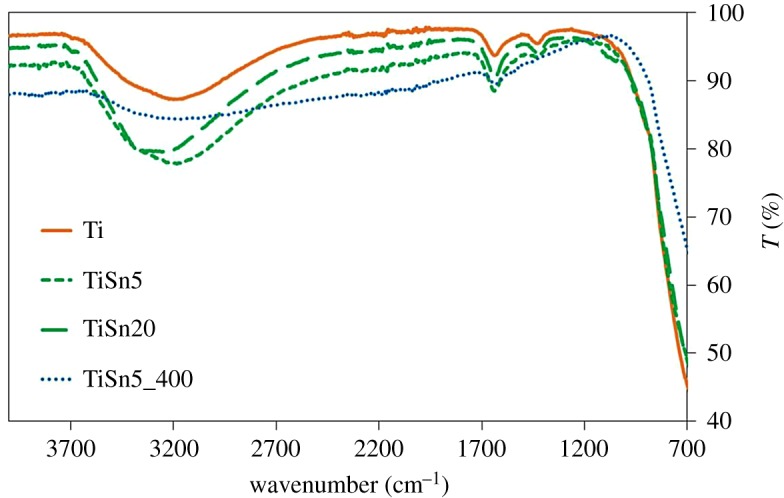
FTIR spectra of the prepared samples.

Figure 5 shows the ATR-FTIR spectra of the prepared materials. With the exception of the calcined sample, all oxides exhibit components at *ca* 1440, 1340 and 1215 cm^−1^ (electronic supplementary material, figure S5), which can be ascribed to NH_4_^+^ species coordinated to different kinds of surface acid sites [36,37]. This evidence further confirms the preferential surface location of N species in Ti and TiSn*x* samples, which can be favoured in the Sn-promoted samples by the acidic sites induced by the presence of SnO_2_ dispersed at the TiO_2_ surface [14]. On the other hand, in the case of the calcined sample, thanks to the high-temperature treatment, the N guest species are able to diffuse into the oxide bulk, giving rise to the colour centres observed in DRS.

FTIR spectra (figure 5) of Ti and TiSn*x* samples also show a broad and intense band in the 3600−3000 cm^−1^ range, characteristic of the vibrational stretching mode of surface OH groups mutually interacting by hydrogen bonding [38], which can partially overlap with the N–H stretching (3500–3300 cm^−1^). The intensity of these bands goes hand in hand with that of the sharper peak around 1635 cm^−1^, ascribable to the in-plane H–O–H bending mode of undissociated water [38]. By comparing this set of samples with the calcined sample, the role of low-temperature treatment in promoting the material surface hydroxylation is evident. The TiSn5_400 sample presents much less intense peaks referred to OH groups and water, in full agreement with XPS O 1s findings.

**Figure 6.**
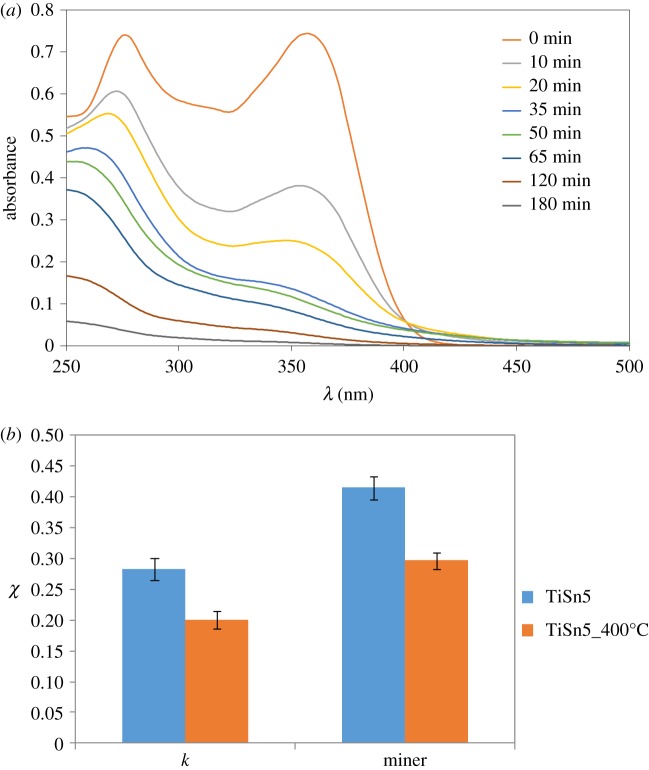
(*a*) UV–vis spectra of TC solution as a function of irradiation time during photocatalytic tests; (*b*) comparison of the photocatalytic activity of TiO_2_/SnO_2_ composites with same Sn content and different heat treatment method: pseudo first-order kinetic constant (×10^−3^ min^−1^) and mineralization degree, normalized by the photocatalyst surface area.

The photocatalytic activity of the samples was tested towards the degradation of tetracycline in water under UV irradiation. Tetracycline was selected because it is considered an emerging pollutant due to its widespread use as antibiotic and possible long-term effects such as antibiotic resistance, endocrine disruption and toxicity on living organisms [39]. Figure 6*a* reports a representative example of the TC solution UV–vis spectra as a function of irradiation time during photocatalytic tests. UV–vis spectra show the progressive disappearance of both the peaks at 270 and 358 nm, without the accumulation of persistent intermediates, as confirmed by the high final mineralization degrees (greater than 60%). The photocatalytic degradation mechanism of tetracycline has been previously investigated by us [4,5], showing comparable oxidation pathways for both pristine TiO_2_ and TiO_2_/SnO_2_ composites. In both cases, the parallel disappearance of the absorption peaks at *ca* 358 and 270 nm was observed, involving initial dehydroxylations, loss of the -N(CH_3_)_2_ group and formation of a carboxylic termination [40]. Figure 6*b* compares the photocatalytic activity of the two 5% Sn containing samples in terms of both molecule disappearance, as initial pseudo first-order rate constant, and final mineralization degree. The calcined sample shows a lower performance in terms of both TC disappearance and mineralization. The much larger anatase content and the promoted surface hydration of the TiSn5 sample might play a beneficial role on its photocatalytic activity.

## 4. Conclusion

A prolonged autoclave treatment at a low temperature was adopted to grow TiO_2_/SnO_2_ precursors prepared by a sol–gel reaction. The hydrothermal conditions appear to promote an enrichment in anatase TiO_2_, more so in the presence of small (5%) Sn amounts. Larger Sn contents (20%), instead, supported the formation of rutile TiO_2_ as the main component, although no crystalline phases related to Sn oxides can be appreciated in diffractograms. The surface localization of finely dispersed SnO_2_, with grain sizes below the threshold of XRPD detectability, was instead supported by XPS determinations. Furthermore, the surface of the composites becomes highly hydrophilic as apparent from both FTIR and XPS determinations.

The possibility to tune at low temperature the TiO_2_ phase composition while maintaining the surface highly hydrophilic is highly desirable in several applications of TiO_2_/SnO_2_ composites, ranging from photocatalysis (as here reported) to more innovative applications, such as in biomaterials. In this respect, the application of TiO_2_/SnO_2_ heterojunctions has been recently proposed in titanium bone implants [41]: in this application, the highly hydrophilic surface would promote cell attachment and bone mineralization.

## Supplementary Material

Experimental data and data analysis

## Data accessibility

Supporting data for this article have been uploaded as part of the electronic supplementary material.

## References

[1] An H, Cui H, Zhou D, Tao D, Li B, Zhai J, Li Q 2013 Synthesis and performance of Pd/SnO_2_–TiO_2_/MWCNT catalysts for direct formic acid fuel cell application. Electrochim. Acta 92, 176–182. (10.1016/j.electacta.2012.12.111)

[2] Kelp G et al. 2016 Self-assembled SnO_2_ micro- and nanosphere-based gas sensor thick films from an alkoxide-derived high purity aqueous colloid precursor. Nanoscale 8, 7056–7067. (10.1039/C5NR07942J) 26960813

[3] Li F, Gao X, Wang R, Zhang T, Lu G 2017 Study on TiO_2_-SnO_2_ core-shell heterostructure nanofibers with different work function and its application in gas sensor. Sensors Actuat. B Chem. 248, 812–819. (10.1016/j.snb.2016.12.009)

[4] Li X, Gao C, Duan H, Lu B, Wang Y, Chen L, Zhang Z, Pan X, Xie E 2013 High-performance photoelectrochemical-type self-powered UV photodetector using epitaxial TiO_2_/SnO_2_ branched heterojunction nanostructure. Small 9, 2005–2011. (10.1002/smll.201202408) 23281221

[5] Rimoldi L, Pargoletti E, Meroni D, Falletta E, Cerrato G, Turco F, Cappelletti G 2018 Concurrent role of metal (Sn, Zn) and N species in enhancing the photocatalytic activity of TiO_2_ under solar light. Catal. Today 313, 40–46. (10.1016/j.cattod.2017.12.017)

[6] Anthony JW, Bideaux RA, Bladh KW, Nichols MC (eds) 1997 Handbook of mineralogy (volume 3). Chantilly, VA: Mineralogical Society of America.

[7] Hou X, Wang X, Liu B, Wang Q, Wang Z, Chen D, Shen G 2014 SnO_2_@TiO_2_ heterojunction nanostructures for lithium-ion batteries and self-powered UV photodetectors with improved performances. ChemElectroChem 1, 108–115. (10.1002/celc.201300053)

[8] Choi S-W, Katoch A, Kim J-H, Kim SS 2015 Striking sensing improvement of n-type oxide nanowires by electronic sensitization based on work function difference. J. Mater. Chem. C 3, 1521–1527. (10.1039/C4TC02057J)

[9] Floriano EA, Scalvi LVA, Saeki MJ, Sambrano JR 2014 Preparation of TiO_2_/SnO_2_ thin films by sol–gel method and periodic B3LYP simulations. J. Phys. Chem. A 118, 5857–5865. (10.1021/jp411764t) 24824227

[10] Marchiori C et al 2014 Unraveling the cooperative mechanism of visible-light absorption in bulk N,Nb codoped TiO_2_ powders of nanomaterials. J. Phys. Chem. C 118, 24 152–24 164. (10.1021/jp507143z)

[11] Tangale NP, Niphadkar PS, Samuel V, Deshpande SS, Joshi PN, Awate SV 2016 Synthesis of Sn-containing anatase (TiO_2_) by sol-gel method and their performance in catalytic water splitting under visible light as a function of tin content. Mater. Lett. 171, 50–54. (10.1016/j.matlet.2016.02.055)

[12] Jia C, Dong T, Li M, Wang P, Yang P 2018 Preparation of anatase/rutile TiO_2_/SnO_2_ hollow heterostructures for gas sensor. J. Alloys Compd. 769, 521–531. (10.1016/j.jallcom.2018.08.035)

[13] Chen H, Liu Y, Wu H, Xiong X, Pan J 2016 FIB-tomographic studies on chemical vapor deposition grown SnO_2_ nanowire arrays on TiO_2_ (001). Mater. Res. Express 3, 125016 (10.1088/2053-1591/3/12/125016)

[14] Guimon C, Gervasini A, Auroux A 2001 XPS study of the adsorption of SO_2_ and NH_3_ over supported tin dioxide catalysts used in de-NOx catalytic reaction. J. Phys. Chem. B 105, 10 316–10 325. (10.1021/jp0108869)

[15] Hellstern HL, Bremholm M, Mamakhel A, Becker J, Iversen BB 2016 Hydrothermal synthesis of TiO_2_@SnO_2_ hybrid nanoparticles in a continuous-flow dual-stage reactor. ChemSusChem 9, 532–539. (10.1002/cssc.201501199) 26822385

[16] Tian Q, Yan J, Yang L, Chen J 2018 Fabrication of three-dimensional carbon coating for SnO_2_/TiO_2_ hybrid anode material of lithium-ion batteries. Electrochim. Acta 282, 38–47. (10.1016/j.electacta.2018.04.044)

[17] Zhang J, Ur Rahman Z, Zheng Y, Zhu C, Tian M, Wang D 2018 Nanoflower like SnO_2_-TiO_2_ nanotubes composite photoelectrode for efficient photocathodic protection of 304 stainless steel. Appl. Surf. Sci. 457, 516–521. (10.1016/j.apsusc.2018.06.307)

[18] Ding J, Huang Z, Zhu J, Kou S, Zhang X, Yang H 2016 Low-temperature synthesis of high-ordered anatase TiO_2_ nanotube array films coated with exposed {001} nanofacets. Sci. Rep. 5, 17773 (10.1038/srep17773) PMC4669522 26634815

[19] Pan X, Zhang N, Fu X, Xu Y-J 2013 Selective oxidation of benzyl alcohol over TiO_2_ nanosheets with exposed {001} facets: catalyst deactivation and regeneration. Appl. Catal. A Gen. 453, 181–187. (10.1016/j.apcata.2012.12.023)

[20] Yang HG, Sun CH, Qiao SZ, Zou J, Liu G, Smith SC, Cheng HM, Lu GQ 2008 Anatase TiO_2_ single crystals with a large percentage of reactive facets. Nature 453, 638–641. (10.1038/nature06964) 18509440

[21] Roy N, Sohn Y, Pradhan D 2013 Synergy of low-energy {101} and high-energy {001} TiO_2_ crystal facets for enhanced photocatalysis. ACS Nano 7, 2532–2540. (10.1021/nn305877v) 23448713

[22] Lazar M, Varghese S, Nair S 2012 Photocatalytic water treatment by titanium dioxide: recent updates. Catalysts 2, 572–601. (10.3390/catal2040572)

[23] Rimoldi L, Meroni D, Cappelletti G, Ardizzone S 2017 Green and low cost tetracycline degradation processes by nanometric and immobilized TiO_2_ systems. Catal. Today 281, 38–44. (10.1016/j.cattod.2016.08.015)

[24] Cai X, Wang C, Chen Y, Cheng Z, Shu R, Zhang J, Bu E, Liao M, Song Q 2018 A novel approach for enhancing hydrogen production from bio-glycerol photoreforming by improving colloidal dispersion stability. Sci. Total Environ. 627, 1464–1472. (10.1016/j.scitotenv.2018.02.009) 30857108

[25] Mamaghani AH, Haghighat F, Lee C 2017 Photocatalytic oxidation technology for indoor environment air purification: the state-of-the-art. Appl. Catal. B Environ. 203, 247–269. (10.1016/j.apcatb.2016.10.037)

[26] Lu J, Huang T, Liu Z, Zhang X, Xiao R 2018 Long-term wettability of titanium surfaces by combined femtosecond laser micro/nano structuring and chemical treatments. Appl. Surf. Sci. 459, 257–262. (10.1016/j.apsusc.2018.08.004)

[27] Ziarati A, Badiei A, Luque R 2018 Black hollow TiO_2_ nanocubes: advanced nanoarchitectures for efficient visible light photocatalytic applications. Appl. Catal. B Environ. 238, 177–183. (10.1016/j.apcatb.2018.07.020)

[28] Jensen GV, Bremholm M, Lock N, Deen GR, Jensen TR, Iversen BB, Niederberger M, Pedersen JS, Birkedal H 2010 Anisotropic crystal growth kinetics of anatase TiO_2_ nanoparticles synthesized in a nonaqueous medium. Chem. Mater. 22, 6044–6055. (10.1021/cm100469y)

[29] Choi M, Lim J, Baek M, Choi W, Kim W, Yong K 2017 Investigating the unrevealed photocatalytic activity and stability of nanostructured brookite TiO_2_ film as an environmental photocatalyst. ACS Appl. Mater. Interfaces 9, 16 252–16 260. (10.1021/acsami.7b03481) 28459533

[30] Boiadjieva T, Cappelletti G, Ardizzone S, Rondinini S, Vertova A 2003 Nanocrystalline titanium oxide by sol–gel method: the role of the solvent removal step. Phys. Chem. Chem. Phys. 5, 1689–1694. (10.1039/b300791j)

[31] Rimoldi L, Meroni D, Falletta E, Ferretti AM, Gervasini A, Cappelletti G, Ardizzone S 2017 The role played by different TiO_2_ features on the photocatalytic degradation of paracetamol. Appl. Surf. Sci. 424, 198–205. (10.1016/j.apsusc.2017.03.033)

[32] Szuber J, Czempik G, Larciprete R, Koziej D, Adamowicz B 2001 XPS study of the L-CVD deposited SnO_2_ thin films exposed to oxygen and hydrogen. Thin Solid Films 391, 198–203. (10.1016/S0040-6090(01)00982-8)

[33] Benkoula S et al 2015 Water adsorption on TiO_2_ surfaces probed by soft X-ray spectroscopies: bulk materials vs. isolated nanoparticles. Sci. Rep. 5, 15088 (10.1038/srep15088) 26462615 PMC4604456

[34] Asahi R, Morikawa T, Irie H, Ohwaki T 2014 Nitrogen-doped titanium dioxide as visible-light-sensitive photocatalyst: designs, developments, and prospects. Chem. Rev. 114, 9824–9852. (10.1021/cr5000738) 25216232

[35] Meroni D, Ardizzone S, Cappelletti G, Oliva C, Ceotto M, Poelman D, Poelman H 2011 Photocatalytic removal of ethanol and acetaldehyde by N-promoted TiO_2_ films: the role of the different nitrogen sources. Catal. Today 161, 169–174. (10.1016/j.cattod.2010.08.013)

[36] Murcia JJ, Hidalgo MC, Navío JA, Araña J, Doña-Rodríguez JM 2013 In situ FT-IR study of the adsorption and photocatalytic oxidation of ethanol over sulfated and metallized TiO_2_. Appl. Catal. B Environ. 142–143, 205–213. (10.1016/j.apcatb.2013.05.022)

[37] Michalow-Mauke KA, Lu Y, Kowalski K, Graule T, Nachtegaal M, Kröcher O, Ferri D 2015 Flame-made WO_3_/CeO_x_-TiO_2_ catalysts for selective catalytic reduction of NO_x_ by NH_3_. ACS Catal. 5, 5657–5672. (10.1021/acscatal.5b01580) 26668943

[38] Meroni D, Lo Presti L, Di Liberto G, Ceotto M, Acres RG, Prince KC, Bellani R, Soliveri G, Ardizzone S 2017 A close look at the structure of the TiO_2_-APTES interface in hybrid nanomaterials and its degradation pathway: an experimental and theoretical study. J. Phys. Chem. C 121, 430–440. (10.1021/acs.jpcc.6b10720) PMC5295244 28191270

[39] Homem V, Santos L 2011 Degradation and removal methods of antibiotics from aqueous matrices – a review. J. Environ. Manage. 92, 2304–2347. (10.1016/j.jenvman.2011.05.023) 21680081

[40] Cao M, Wang P, Ao Y, Wang C, Hou J, Qian J 2016 Visible light activated photocatalytic degradation of tetracycline by a magnetically separable composite photocatalyst: graphene oxide/magnetite/cerium-doped titania. J. Colloid Interface Sci. 467, 129–139. (10.1016/j.jcis.2016.01.005) 26799623

[41] Zhou R et al 2018 Enhanced osseointegration of hierarchically structured Ti implant with electrically bioactive SnO_2_–TiO_2_ bilayered surface. ACS Appl. Mater. Interfaces 10, 30 191–30200. (10.1021/acsami.8b10928) 30130089

