# Supplementary material for: Role of the growth step on the structural, optical and surface features of TiO2/SnO2 composites
Source: R Soc Open Sci. 2019 Jan 9;6(1):181662. doi: 10.1098/rsos.181662 (PMC6366208; doi:10.1098/rsos.181662)
Supplement: Experimental data and data analysis [file rsos181662supp1.docx]

**Role of the growth step on the structural, optical and surface features of TiO_2_/SnO_2_ composites**

Luca Rimoldi^a,b^, Daniela Meroni^a,b,*^, Eleonora Pargoletti^a,b^, Iolanda Biraghi^a^, Giuseppe Cappelletti^a,b^, Silvia Ardizzone^a,b^

*^a^ Dipartimento di Chimica, Università degli Studi di Milano, Via Golgi 19, 20133 Milano, Italy*

*^b^ Consorzio Interuniversitario Nazionale per la Scienza e la Tecnologia dei Materiali (INSTM), Via Giusti 9, 50121 Firenze, Italy*

*^*^Corresponding authors:* [*daniela.meroni@unimi.it*](mailto:daniela.meroni@unimi.it)

**Electronic supplementary material**

**S1. XPS results**

**a)**

**b)**

Figure S1 – XPS survey spectra: TiSn5 (a) and TiSn5_400 (b).

Table S1 – XPS O 1s fitting parameters.

| Sample | peak position (eV) | FWHM (eV) | area |
| --- | --- | --- | --- |
| TiSn5 | 529.5 | 1.4 | 256 |
|  | 530.7 | 1.5 | 197 |
|  | 531.9 | 1.8 | 230 |
| TiSn5_400 | 529.4 | 1.4 | 382 |
|  | 530.5 | 1.4 | 165 |
|  | 531.9 | 1.7 | 82 |

**S2. EDX results**


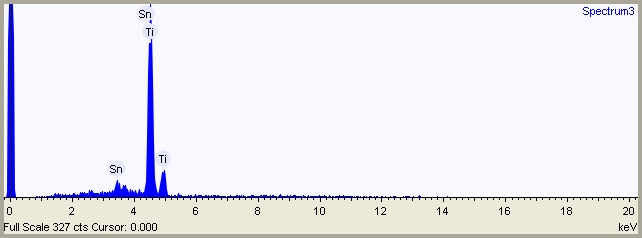

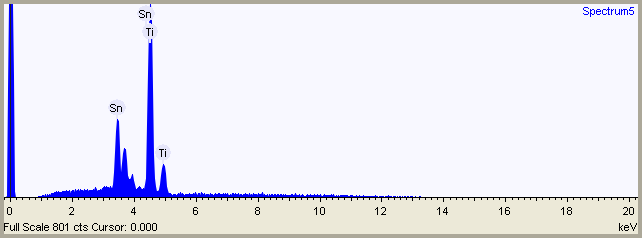


**b)**

**a)**


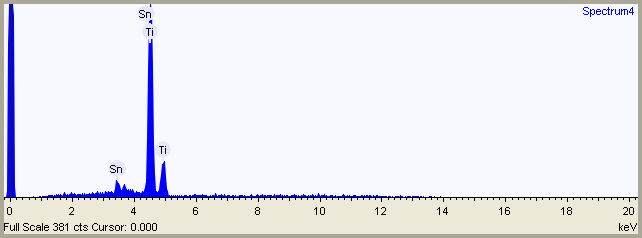


**c)**

Figure S2 – Representative examples of EDX curves of TiSn5 (a), TiSn20 (b), and TiSn5_400 (c).

Table S2 – EDX results.

| sample | Ti %_w_ | Sn %_w_ |
| --- | --- | --- |
| TiSn5 | 91.0 ± 0.5 | 9.1 ± 0.5 |
| TiSn20 | 63.3 ± 0.3 | 36.7 ± 0.3 |
| TiSn5_400 | 91.2 ± 0.8 | 8.8 ± 0.8 |

**S3. BET results**

Figure S3 – Pore size distributions.

**S4. DRS results**

Figure S4 – Kubelka-Munk plot.

**S5. FTIR results**

Figure S5 – FTIR spectra (900-1900 cm^-1^ region) of TiSn20.
